# Tripartite Interaction of Epigenetic Regulation, Brain Aging, and Neuroinflammation: Mechanistic Insights and Therapeutic Implications

**DOI:** 10.3390/epigenomes9040038

**Published:** 2025-10-05

**Authors:** Shenghui Mi, Hideyuki Nakashima, Kinichi Nakashima

**Affiliations:** Department of Stem Cell Biology and Medicine, Graduate School of Medical Sciences, Kyushu University, 3-1-1, Maidashi, Higashi-ku, Fukuoka 812-8582, Japan; mi.shenghui.212@s.kyushu-u.ac.jp

**Keywords:** epigenetics, neuroinflammation, brain aging, endogenous DNA ligands, DNA sensor, neurodegenerative disease

## Abstract

Aging of the central nervous system (CNS) involves widespread transcriptional and structural remodeling, prominently marked by synaptic loss, impaired neurogenesis, and glial dysfunction. While age-related gene expression changes have been documented for decades, recent genome-wide next-generation sequencing studies emphasize the importance of epigenetic mechanisms—such as DNA methylation and histone modification—in shaping these profiles. Notably, these modifications are potentially reversible, making them promising targets for therapeutic intervention. However, the mechanisms by which age-associated factors, such as inflammation and oxidative stress, orchestrate these epigenetic alterations across distinct CNS cell types remain poorly understood. In this review, we propose a framework for understanding how aging and neuroinflammation are regulated by epigenetic mechanisms, contributing to brain dysfunction and disease vulnerability.

## 1. Introduction

Since August Weismann’s seminal exploration into the nature of death in 1891 [1], our understanding of aging has progressively deepened alongside advances in experimental techniques and analytical tools. Aging is now widely characterized by several hallmarks: genomic instability, telomere attrition, epigenetic alterations, loss of proteostasis, impaired autophagy, deregulated nutrient sensing, mitochondrial dysfunction, cellular senescence, stem cell exhaustion, altered intercellular communication, chronic inflammation, and dysbiosis [2].

Brain aging is the primary risk factor for neurodegenerative diseases such as Alzheimer’s disease (AD) and Parkinson’s disease (PD); these disorders are characterized by chronic neuroinflammation, misfolded-protein aggregation (amyloid-β (Aβ)/tau in AD; α-synuclein (α-syn) in PD), progressive synaptic and circuit dysfunction, and ultimately selective neuronal loss.

**Figure 1:**
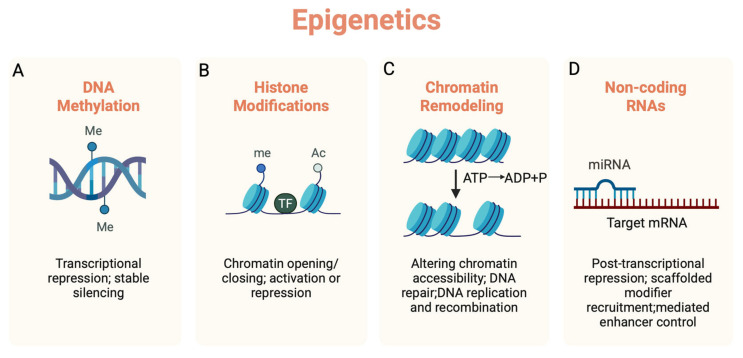
Four major epigenetic mechanisms regulating gene expression. (**A**) DNA methylation at CpG sites generally leads to transcriptional repression and stable silencing of genes. (**B**) Histone modifications (e.g., acetylation and methylation) modulate the opening/closing of nucleosomes, resulting in the activation or repression of genes. (**C**) Regulation of gene expression by altering the position and structure of nucleosomes and the covalent modifications of histones. This facilitates the binding of transcription factors to DNA, thereby promoting or inhibiting gene transcription. (**D**) non-coding RNAs (e.g., miRNAs) impose post-transcriptional repression, driving mRNA decay/translational inhibition. Me: DNA methylation, Ac: Histone acetylation, me: Histone methylation. Created with BioRender.com.

In both normal brain aging and neurodegenerative diseases, numerous studies have reported the presence of neuroinflammation and specific epigenetic alterations. Epigenetics refers to the regulation of gene expression through modifications that do not alter the underlying DNA sequence. The major epigenetic mechanisms include DNA methylation, histone modifications, non-coding RNAs (ncRNAs) and chromatin remodeling. These modifications can either promote (e.g., histone acetylation) or repress gene expression (e.g., DNA methylation and microRNA (miRNA) mediated suppression of mRNA) (Figure 1).

While extensive efforts have been made to elucidate the relationship between epigenetic modifications and brain aging [3], as well as between neuroinflammation and aging [4], and between epigenetic regulation and neuroinflammatory responses [5], these factors are often examined in isolation. Relatively few studies have integrated all three—epigenetic alterations, brain aging, and neuroinflammation—into a unified framework.

In this review, we aim to bridge this gap by summarizing advances related to each of these processes and exploring their potential interplay. By doing so, we aim to provide new insights into how these interconnected mechanisms collectively shape the aging brain and to highlight their potential implications for therapeutic applications.

## 2. The Role of Epigenetics in Brain Aging

The adult CNS includes neurons, glial cells (astrocytes, oligodendrocytes, microglia), and neural stem cells (NSCs), which are confined to restricted niches. Initially, brain aging was primarily attributed to functional impairments of neurons, which were believed to underlie age-related cognitive deficits. However, recent studies have shown that brain aging is not limited to neurons; glial cells and NSCs also exhibit age-related changes that impair brain functions.

On the other hand, it has been known that cellular aging within the CNS is governed by both extrinsic factors (such as systemic or microenvironmental influences) and intrinsic mechanisms, including epigenetic alterations. In this section, we aim to summarize current knowledge on the roles of epigenetic alterations in the aging processes of each CNS cell type (Table 1).

### 2.1. Neural Stem Cell Aging

NSCs can give rise to neurons and glial cells (astrocytes and oligodendrocytes, but not microglia, which originate from yolk sac-derived hematopoietic progenitors) [17]. Once thought to be absent in the adult brain, NSCs were identified in the subgranular zone (SGZ) of the hippocampal dentate gyrus and subventricular zone (SVZ) of the lateral ventricles [18,19,20]. Subsequent studies have demonstrated that during aging and in various neurodegenerative diseases such as AD and PD, there is an accumulation of quiescent NSCs accompanied by a reduction in proliferative and neurogenic capacities [21]. These findings underscore the importance of maintaining cell cycle homeostasis of NSCs in preventing age-associated cognitive decline.

Epigenetic regulation plays a critical role in NSC maintenance and differentiation throughout life. Among these mechanisms, nuclear architecture alterations have emerged as key contributors to NSC aging. LaminB1, a major component of the nuclear lamina, anchors chromatin domains known as lamina-associated domains (LADs), which are typically enriched with repressive histone marks such as histone H3 Lysine 9 di- or tri-methylation (H3K9me2/3) and DNA methylation [22]. In aging hippocampal NSCs, LaminB1 expression progressively declines, leading to the disruption of LADs and global chromatin decondensation [6,7]. This structural alteration results in the silencing of neurogenic genes, thereby impairing NSC function to produce neurons. Importantly, ectopic expression of LaminB1 in aged NSCs has been shown to restore their proliferative and neurogenic capacity in vitro [6].

Histone methylation also plays a dynamic role in regulating the transition of NSCs between quiescent and activated states. It has been reported that loss of the H3K4 methyltransferase SET domain containing 1A (Setd1a) promotes NSC activation and enhances neurogenesis [8], whereas a decrease in SET domain containing 8 (Setd8) expression, the methyltransferase responsible for H4K20me1, leads to the upregulation of quiescence-associated genes, leading to NSC aging [9]. These findings suggest that histone modification patterns are not merely markers but active drivers of age-related transcriptional programs in NSCs.

Central to active DNA demethylation is the Ten-Eleven Translocation (TET) family of enzymes, which catalyze the conversion of 5-methylcytosine (5mC) to 5-hydroxymethylcytosine (5hmC). In the aged mouse hippocampal NSCs, *Tet2* expression is significantly reduced, impairing adult neurogenesis, while its overexpression can restore cognitive function [10], probably due to the recovery of neurogenesis. These data indicate that TET-dependent demethylation functions as a mechanistic lever linking epigenome remodeling to NSC state and aging.

Overall, the current evidence supports the necessity of epigenetic regulation to enhance NSC activity as a potential strategy against aging and neurodegeneration.

### 2.2. Neuronal Aging

Neurons are the fundamental units of cognitive function in the brain, and their aging is considered a central driver of global brain aging and cognitive decline. Aged neurons exhibit multifaceted structural and functional deterioration, including impaired synaptic plasticity, reduced responsiveness to ion channels, and disrupted metabolic homeostasis [23]. One of the most striking morphological hallmarks is the loss of dendritic arbors and dendritic spines, which compromises integrative synaptic capacity and impairs learning and memory. These structural elements rely heavily on a well-organized cytoskeleton, particularly the microtubule and actin networks. During aging, α-tubulin undergoes hyperacetylation—a cytoskeletal modification associated with neuronal senescence [11]. While this phenomenon has been well documented, the upstream epigenetic mechanisms regulating cytoskeletal remodeling in aging neurons remain poorly understood and warrant further investigation.

At the molecular level, DNA methylation serves as a key marker of the epigenetic clock. The epigenetic clock model demonstrates a high concordance between DNA methylation age and chronological age in neural tissues, with aged brains frequently exhibiting accelerated epigenetic aging [24]. Notably, expression of calcium-signaling–related gene *EF-Hand Calcium Binding Domain 5* (*EFCAB5*) in brain regions such as the cerebellum and prefrontal cortex is negatively correlated with epigenetic age acceleration, suggesting that methylation-mediated repression of functionally critical genes may contribute to cognitive decline [24].

Histone modifications, particularly acetylation, constitute another major axis of epigenetic regulation. Histone deacetylases (HDACs) remove acetyl groups from histone tails, promoting chromatin compaction and gene silencing. Increased expression of Hdac2 has been observed in aging neurons [25]. This upregulation represses the transcription of key synaptic genes, including *Brain-derived neurotrophic factor* (*Bdnf*) and *Early growth response-1* (*Egr1*), thereby contributing to synaptic loss and reduced plasticity [12]. Notably, selective HDAC inhibitors—including SAHA (vorinostat) and sodium butyrate—can reverse these transcriptional deficits and ameliorate memory impairment. In parallel, Hdac1 plays a non-redundant role in maintaining genomic stability through DNA repair pathways, and its dysfunction has been linked to neurodegeneration [26].

In summary, DNA methylation and HDAC-driven histone deacetylation collectively form a core epigenetic network underpinning neuronal aging. These regulators not only reflect cellular aging status but also serve as potential therapeutic targets for mitigating neurodegeneration and cognitive decline.

### 2.3. Astrocytic Aging

As the most abundant glial cell type in the CNS, astrocytes play critical roles in supporting neuronal function through synaptic modulation, neurotransmitter recycling, metabolic coupling, and blood–brain barrier (BBB) regulation. With aging, astrocytes undergo functional changes, including reduced glutamate uptake, pro-inflammatory activation, and metabolic reprogramming [27]. These alterations not only reflect intrinsic cellular senescence but also actively contribute to age-related cognitive decline and neurodegeneration.

The role of epigenetic mechanisms in astrocyte differentiation has been extensively characterized. Specifically, DNA demethylation of astrocyte-specific genes is a critical regulatory switch for astrocyte lineage commitment during brain development [28,29,30]. By contrast, in the context of astrocyte aging, chromatin profiling indicates a shift toward a more repressive landscape, with a decrease in the active mark H3K4me3 and a concomitant increase in the repressive mark H3K9me3 at functionally relevant loci [31]. On the other hand, emerging evidence from AD models has implicated histone deacetylases in age-related astroglial dysfunction. For example, increased expression of HDAC7 has been observed in both AD patient brain tissue and PS19 tauopathy mouse models, where it contributes to tau accumulation and neurodegeneration [13]. Notably, targeted inhibition of Hdac7 restored cognitive function in PS19 mice, highlighting a potential therapeutic entry point for epigenetic modulation of aging astrocytes [13].

Taken together, elucidating the epigenetic alterations that underlie astrocyte senescence holds significant therapeutic promise. Decoding the regulatory layers that govern age-associated astroglial reprogramming may unveil novel molecular targets for mitigating astrocyte-driven neuroinflammatory cascades and cognitive decline during brain aging.

### 2.4. Oligodendrocytic Aging

Oligodendrocytes are the primary myelin-producing cells in the CNS, and their long-term function relies on stable epigenetic maintenance. A methylome analysis of human frontal white matter revealed a strong correlation between chronological and epigenetic age (R^2^ ≈ 0.99, *p* < 10^−14^) in both bulk tissue and directly converted oligodendrocytes [32], underscoring their dominant role in white matter methylome aging.

Epigenetic analysis of aged rodent brains revealed a marked decrease in transcriptional repression characterized by reduced histone methylation and deacetylation. Concurrently, enhanced histone acetylation and chromatin accessibility were observed [33]. These changes coincide with the downregulation of oligodendrocyte-related genes, including *SRY-box transcription factor 10* (*Sox10*) and *Oligodendrocyte transcription factor 2* (*Olig2*), and an aberrant increase in SRY-box transcription factor 2 (*Sox2*) positive cells within mature oligodendrocyte populations, indicative of lineage fluctuation [33]. In another transcriptomic study focused on oligodendrocyte precursor cells (OPCs), global DNA hypomethylation was observed in 16-month-old rats, which was associated with reduced expression of *DNA methyltransferase 1* (*Dnmt1*) [14]. Collectively, these findings suggest that age-associated erosion of epigenetic memory impairs oligodendrocyte identity and compromises myelin maintenance.

These epigenetic alterations may contribute to white matter degeneration in aging and neurodegenerative conditions such as AD and multiple sclerosis. Recent findings demonstrate that pharmacological inhibition of epigenetic silencing using a small-molecule epigenetic-silencing-inhibitor (ESI1) significantly enhances remyelination and ameliorates cognitive deficits in aged mice [34]. These results underscore the therapeutic potential of reprogramming the oligodendroglial epigenetic landscape to rejuvenate regenerative capacity in the aging brain.

### 2.5. Microglial Aging

Age-associated epigenetic remodeling in microglia facilitates a phenotypic shift from a homeostatic state to a chronically primed, pro-inflammatory profile, marked by reduced activation thresholds and impaired resolution capacity. High-resolution Assay for Transposase-Accessible Chromatin using sequencing (ATAC-seq) profiling across young (3 months), middle-aged (14 months), and aged (24 months) mouse microglia revealed widespread alterations in chromatin accessibility during aging [35]. Promoter-proximal regions of positively age-dependent microglia genes, including *AXL Receptor Tyrosine Kinase* (*Axl*), *Cluster of Differentiation 74* (*Cd74*), *secreted phosphoprotein 1* (*Spp1*), and *Cystatin F* (*Cst7*), became increasingly accessible with age, suggesting epigenetic priming of inflammatory pathways; conversely, loci associated with negatively age-dependent microglia genes exhibited decreased accessibility [35]. Motif analysis further indicates an age-linked gain of CCAAT/enhancer binding protein beta (C/ebpβ) and a loss of Myocyte Enhancer Factor 2C (Mef2c) activity [35]. In parallel, an independent lifespan single-cell atlas corroborates age-linked state transitions and reduced network plasticity [36]. In line with the priming concept, longitudinal studies in the chronic unpredictable mild stress (CUMS) paradigm demonstrate that microglial activation follows state-dependent dynamics: within 2–4 weeks after CUMS initiation, re-exposure to acute stress modulates activation markers (enhancing or reversing them). In contrast, microglia in the primed state after 6 weeks become unresponsive, suggesting a transition from maladaptive hyperactivation to dysfunction [37]. This provides indirect evidence that microglial immune function becomes progressively dysregulated in a chronic inflammatory milieu.

*Sirtuin 1* (*Sirt1*) expression is downregulated in aged microglia, leading to elevated *Interleukin-1 beta* (*Il-1β*) expression and exacerbation of age-related memory deficits in mouse models [15]. Mechanistically, because Sirt1 enhances Dnmt1 activity through deacetylation, Sirt1 reduction inactivates Dnmt1, promoting demethylation at the proximal promoter of *Il-1β* and thereby derepressing its transcription [15]. Notably, pharmacological blockade of Il-1β signaling ameliorates cognitive impairment in AD mouse models, emphasizing the pathological relevance of this epigenetically regulated inflammatory axis [38].

Beyond static remodeling, microglia acquire innate immune memory (training or tolerance) through epigenetic reprogramming that shapes subsequent responses to pathology [39]. Environment-dependent enhancer maps in human microglia and conserved epigenomic signals across mouse–human datasets link inflammatory activation to disease-relevant regulatory programs [40,41]. Functionally, aged brains accumulate lipid-droplet microglia with defective phagocytosis and heightened reactive oxygen species (ROS]/cytokine output, further coupling epigenetic drift to persistent inflammation [42]. DNA demethylation pathways (e.g., TET2) also tune microglial activation in neurodegenerative contexts, highlighting multi-layered epigenetic control over age-biased immune phenotypes [16].

Collectively, these data support a model in which aging-associated remodeling of microglial chromatin accessibility and histone/DNA-methylation pathways establishes transcriptional priming, reduces plasticity, and sustains neuroinflammation [43].

## 3. The Role of Epigenetics in Neuroinflammation

Neuroinflammation plays a dual role in maintaining brain health. Under physiological conditions, transient inflammatory responses orchestrated by microglia are essential for tissue repair, synaptic remodeling, and clearance of pathogens or damaged neurons. However, persistent or excessive activation of microglia shifts this protective response toward a chronic inflammatory state that contributes to synaptic dysfunction, neuronal loss, and ultimately cognitive decline [44,45]. This maladaptive transformation is increasingly recognized as a central driver of brain aging and neurodegenerative pathologies such as AD, in which aberrant tau accumulation and Aβ deposition both elicit sustained microglial activation and cytokine production [46].

Beyond intrinsic pathological protein aggregates, exogenous oxidative stress can destabilize the brain’s immune equilibrium and promote chronic neuroinflammation. As detailed in Section 3.1 (Oxidative Stress), these stimuli also induce widespread epigenetic alterations in neural cells, including DNA methylation reprogramming, histone acetylation/deacetylation dynamics, and chromatin remodeling, which modulate the inflammatory state of microglia. Furthermore, recent research findings indicate that aging and epigenetic alterations induce genomic DNA (gDNA) damage, release mitochondrial DNA (mtDNA), and generate cDNA transcribed from mRNAs containing retrotransposon elements (such as the long interspersed nuclear element-1 (L1) retrotransposon). These serve as endogenous “danger signals,” activating innate immune pathways through epigenetic regulatory mechanisms.

In this section, we systematically summarize how oxidative stress, endogenous DNA ligands, chromatin remodeling factors, ncRNAs and environmental, lifestyle factors influence neuroinflammation at the epigenetic level. We further address how these epigenetic modifications shape microglial phenotypes, thereby modulating the threshold and trajectory of neuroinflammatory responses in the aging brain.

### 3.1. Oxidative Stress

ROS, a byproduct of aerobic metabolism, must be tightly regulated to minimize cellular toxicity. Mitochondrial electron transport, nicotinamide adenine dinucleotide phosphate oxidase activation, and inflammatory signaling are major sources of ROS in cells. Under physiological conditions, antioxidants such as glutathione and superoxide dismutase maintain redox homeostasis. However, aging, mitochondrial dysfunction, and environmental stress factors such as radiation and heavy metal exposure can cause the accumulation of pathological ROS, leading to oxidative stress.

Beyond causing direct molecular damage, ROS act as potent modulators of the epigenome during neuroinflammation. Oxidative stress during neuroinflammation does not simply cause a global decrease in DNA methylation; rather, it reshapes methylation patterns in a locus- and region-specific manner, partly by modulating the expression and catalytic activity of Dnmts [47,48]. Simultaneously, oxidative stress skews the chromatin-acetylation equilibrium toward deacetylation. Acute ROS exposure increases class I/II HDAC activity and lowers bulk H3/H4 acetylation. In neurons, Hdac2 is recruited to stress-responsive loci and, in part through complex formation with *forkhead box O3a* (*Foxo3a*), enforces transcriptional repression programs that bias cells toward apoptosis [49]. Consistently, active histone marks—H3K9ac and, most notably, H4K12ac—decline in the aged hippocampus, indicating reduced transcriptional plasticity [50].

Furthermore, ROS-driven activation of Nuclear Factor-kappa B (NF-κB) and Mitogen-Activated Protein Kinase cascades sustain inflammatory gene expression in glia, particularly microglia [51]. This effect is compounded by redox-dependent inhibition of Sirt1: oxidative DNA damage activates Poly (ADP-ribose) polymerase 1(Parp-1), depleting nicotinamide adenine dinucleotide (NAD^+^) and thereby lowering the activity of the NAD^+^-dependent deacetylase Sirt1, which normally restrains NF-κB by deacetylating the p65 subunit. The resulting NAD^+^ loss and *Sirt1* downregulation weaken this negative feedback and amplify cytokine production [52,53,54].

In glial cells, such ROS-linked epigenetic effects are closely tied to durable pro-inflammatory programming. Prolonged oxidative stress induces the expression of microRNAs—most notably miR-155, which targets Suppressor of cytokine signaling 1 and reinforces a self-perpetuating inflammatory state; by contrast, miR-146a is often co-induced as a context-dependent feedback regulator of Interleukin-1 Receptor-Associated Kinase 1/Tumor Necrosis Factor Receptor Associated Factor 6/NF-κB signaling in microglia and astrocytes [55,56].

Taken together, ROS acts not only as a mediator of oxidative injury but also as a key epigenetic regulator of neuroimmune activation. This ROS–epigenetic–inflammation axis likely drives the transition from acute responses to chronic neuroinflammation during brain aging and neurodegenerative disease.

### 3.2. Endogenous DNA Ligands

**Figure 2:**
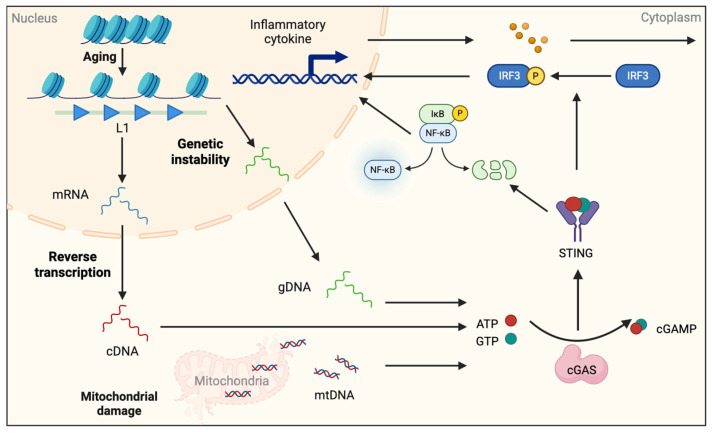
Aging- and epigenetic alteration-induced endogenous DNA fragments trigger cGAS-STING pathway activation. With aging, there is a global increase in histone deacetylation, loss of stability in gene expression, and gDNA damages. Small, damaged fragments of gDNA leak from the nucleus into the cytosol. At the same time, elevated L1 expression leads to an increase in reverse-transcribed DNA from L1 mRNA, resulting in the accumulation of L1 cDNA in the cytosol. Aging-related mitochondrial damage causes mtDNA to leak into the cytosol. These endogenous DNAs activate the cGAS–STING pathway, producing cGAMP. In turn, cGAMP activates the transcription factors IRF3 and NF-κB—via phosphorylation and degradation of IκB, respectively. The activated transcription factors enter the nucleus to drive the transcription of inflammatory cytokines, thereby triggering neuroinflammation. mRNA: messenger RNA. cDNA: complementary DNA. gDNA: genomic DNA. mtDNA: mitochondrial DNA. Created with BioRender.com.

Major sources of endogenous DNA ligands include mitochondrial DNA leakage (for mtDNA), nuclear DNA damage (for gDNA), and retroelement activity (for L1 cDNA). Endogenous DNA ligands in cytosol can be a potent trigger of innate immune response in the CNS: their abnormal accumulation activates the cGAS–STING pathway, driving type-I interferon and NF-κB programs that promote microglial reactivity and neurodegeneration, especially in aging brains [57,58] (Figure 2). L1 expression is normally suppressed by DNA methylation and H3K9me3. However, in aged cells, loss of epigenetic silencing unlocks L1 transcription, leading to the accumulation of L1 cDNA in the cytoplasm following reverse transcription. This also activates the cGAS–STING pathway [59,60]. De Cecco et al. demonstrated that *forkhead box A1* (*FOXA1*) is upregulated in human senescent cells and binds the 5′-UTR of L1, facilitating its reactivation and thereby triggering the cGAS–STING–IFN cascade [61].

Upon sensing endogenous DNA ligands, including L1 cDNA, cGAS synthesizes 2′3′-cGAMP to activate STING, which recruits TANK-binding kinase 1 and phosphorylates Interferon Regulatory Factor 3 (IRF3), while also engaging NF-κB; the result is sustained expression of Interferon beta (IFN-β) and inflammatory cytokines such as Tumor Necrosis Factor alpha (TNF-α) and Interleukin-6 (IL-6) [62]. On the other hand, our previous study has demonstrated that endogenous DNA ligands derived from degenerating neurons activate the Toll-like receptor 9 (TLR9) pathway in microglia within an epileptic mouse model, prompting these cells to release diverse inflammatory cytokines [63]. The effect of these inflammatory cytokines has been reported in brain aging and AD models, where STING activation drives reactive microglial states and cognitive decline, and pharmacological or genetic STING inhibition ameliorates pathology [64,65].

Collectively, an increase in endogenous DNA ligands and the sensing by cGAS–STING form a mechanistic bridge between cellular senescence, chromatin dysregulation, and innate immune activation in the aging brain.

### 3.3. Chromatin Remodeling Factors and ncRNAs

ncRNAs assist in the formation of chromatin structures and participate in the regulation of gene expression. Long ncRNAs (lncRNAs) recruit epigenetic enzymes (e.g., *Polycomb repressive complex 2* (*PRC2*)) to specific DNA sites, altering local histone modifications [66,67,68]. Enhancer RNA (eRNA) promotes transcription by interacting with mediator and cohesin to help enhancers form loops and bind to promoters, thereby inducing the release of RNA polymerase II from its paused state [69,70,71]. miRNAs act indirectly: they lower or raise the expression levels of writers, erasers, and readers (e.g., *Enhancer of zeste homolog 2* (*EZH2*), Lysine Demethylases (KDMs), *HDAC*s, *Bromodomain containing 4* (*BRD4*)) and shift transcription factor (TF) networks, which then affect chromatin openness [72].

These RNAs collectively establish an “epigenetic framework”—balancing DNA methylation, histone modifications, nucleosome placement, and enhancer activity in response to stimuli. They tip inflammatory genes toward either an “activated state” or an “suppressed state.” In microglia, disease- and age-associated lncRNAs (e.g., *Nuclear Enriched Abundant Transcript 1* (*NEAT1*)) function with chromatin regulators of cytokine genes. miR-155 promotes inflammation, while miR-124 and miR-146a suppress it by negatively regulating NF-κB/Signal transducer and activator of transcription/Activator protein 1 signaling [73,74,75,76,77]. Furthermore, it has also been reported that, as a broad mechanism for enhancer activation, RNAPII binding and eRNA synthesis are crucial for gene expression programs responding to neuronal activity, thereby controlling neuronal function [78]. In parallel, CNS cell cultures revealed that Apolipoprotein E (APOE)-activated ncRNA (AANCR), which exhibits eRNA enhancement characteristics, can confer an inflammatory phenotype to astrocytes by influencing APOE expression [43]. In the future, the targeted inhibition of pro-inflammatory ncRNAs may emerge as a reliable therapeutic approach for controlling neuroinflammation [79].

### 3.4. Environmental Factors Affecting the Interaction of Epigenetic Alteration and Inflammation

Recent studies have indicated that environmental factors (such as air pollution, heavy metal contamination, chemical exposure, and maternal-fetal immune environments) and lifestyle habits (including circadian rhythms, exercise, and diet) also modulate neuroinflammation through epigenetic alterations. These findings suggest that the neuroinflammation is not only linked to intrinsic factors within the organism (such as oxidative stress) but also involves individual variations driven by external factors (such as lifestyle habits). In this section, we introduce circadian rhythm disruption, exposure to air pollution and drug (Valproic acid (VPA)) exposure as representative examples of how neuroinflammation is induced through epigenetic alterations.

#### 3.4.1. Circadian Clock

Brain and Muscle ARNT-Like 1 (BMAL1) is a core component of the molecular circadian clock that orchestrates daily rhythms in physiology and behavior. Functioning as a transcription factor, BMAL1 forms a heterodimer with CLOCK, which binds to E-box elements in target gene promoters to drive rhythmic expression of core clock genes such as *Period Circadian Regulator 1* (*PER1*), *Cryptochrome Circadian Regulator 1* (*CRY1*) and *Rev-Erb alpha* (*Rev-erbα*). This transcriptional activation initiates the primary transcriptional–translational feedback loop underlying circadian oscillations. Beyond its canonical role in circadian regulation, BMAL1 exerts pleiotropic effects across multiple biological systems. It influences cellular metabolism, mitochondrial function, oxidative stress responses, and immune regulation. Loss of BMAL1 function in animal models disrupts circadian rhythmicity, accelerates aging phenotypes, and exacerbates neurodegenerative pathology. In the cerebral cortex of Bmal1 knockout mice, expression of the redox genes *NAD(P)H dehydrogenase (quinone 1)* (*Nqo1*) and *Aldehyde dehydrogenase 2 family member* (*Aldh2*) decreased, while the number of activated microglia was increased. This indicates that Bmal1 knockout leads to increased brain vulnerability to oxidative stress. Concurrently, Bmal1^+/−^ heterozygous mice subjected to induced oxidative stress (3-NP treatment) exhibited neurodegenerative pathological features [80]. Furthermore, Bmal1–Rev-erbα axis is a central regulatory pathway of the circadian clock, and knockout of *Rev-Erba* revealed that the Bmal1–Rev-erbα axis critically regulates microglial activation via the NF-κB pathway [81]. Rev-ERBs are known to suppress transcription through multiple mechanisms, including DNA-binding domain (DBD)-dependent binding to Retinoic Acid-Related Orphan Receptors Response Elements (ROREs) [82], tissue-specific transcription factors [83], eRNA expression [84], and DBD-independent interactions regulating chromatin circulation [85]. Although the mechanism by which the Bmal1–Rev-erbα axis regulates transcription of NF-κB pathway-associated genes remains unclear, however, combined with findings from another experiment: 6 h of sleep deprivation affects chromatin accessibility in the mouse cerebral cortex, thereby exerting long-term effects on gene expression [86], it can be inferred that circadian rhythm balance plays a crucial role in regulating neuroinflammation.

#### 3.4.2. PM_2.5_

Persistent epidemiological and experimental studies demonstrate that air pollution (e.g., particulate matter/ozone) is significantly associated with altered DNA methylation patterns, including differential methylation at inflammatory and stress response pathway gene loci [87,88,89]. Particulate matter 2.5 (PM_2.5_) refers to airborne particles with a diameter of less than 2.5 μm. Due to their small size, PM_2.5_ can penetrate deep into the respiratory tract, reach the alveoli, and even enter the bloodstream, thereby exerting systemic effects. In a study related to PM_2.5_, it was reported that inhaling PM_2.5_ (1 and 5 mg/kg bw) every other day for 4 weeks induced neuroinflammation in C57BL/6 mice, impaired synaptic integrity, and further reduced spatial learning and memory abilities [90]. Mechanistically, these effects are mediated via NF-κΒ p65–dependent downregulation of miR-574-5p, which targets *β-secretase* (*Bace1*). Notably, overexpression of miR-574-5p in the hippocampus reduced *Bace1* expression, restored synaptic function, and improved spatial memory and learning following PM_2.5_ exposure [90]. Supporting a translational relevance to humans, genome-wide DNA methylation analysis of prefrontal cortex tissue from 159 AD patients identified 24 CpG sites mediating the association between PM_2.5_ exposure and neuropathological markers, several of which reside within genes associated with neuroinflammation [91]. Collectively, these findings reveal a previously unrecognized link between PM_2.5_-induced air pollution and neuroinflammation in AD, potentially mediated by epigenetic modifications, highlighting the environmental factor’s contributions to the pathogenesis of neurodegenerative diseases.

#### 3.4.3. VPA

VPA is an antiepileptic drug; however also known as an HDAC inhibitor [92]. We have previously shown that VPA enhances neuronal differentiation of NSCs via HDAC inhibitory activity. Furthermore, we have revealed that VPA administration following NSC transplantation into spinal cord-injured mice increases the replenishment of newly generated neurons at the injury site, leading to dramatic recovery of hindlimb function [93]. However, VPA therapy also has adverse effects, and when administered to pregnant mothers, it increases the risk of developing autism spectrum disorder (ASD) [94]. Neuroinflammation is observed in the brains of ASD patients and model mice, and this inflammation is attributable, at least in part, to the pathogenesis of ASD, whereas the cause of the inflammation remains elusive. Nevertheless, it has recently been reported that L1 expression is upregulated in the brain of VPA-exposed ASD model mice compared to controls, probably due to the promotion of histone acetylation of L1 genes through the function of VPA as an HDAC inhibitor [95]. Moreover, VPA has been shown to induce cellular senescence [96], which may shift the epigenetic status of the cells toward aged states, leading to an increase in L1 expression. Therefore, this age-associated epigenetic state shift may also contribute to the enhanced expression of L1 in the VPA-exposed ASD model mice. As explained in Figure 2, L1 is reverse transcribed into cDNA, functioning as an endogenous DNA ligand for DNA sensors, e.g., cGAS and TLR9, inducing neuroinflammation. Taking these facts into consideration, reverse transcriptase inhibitors could be leveraged as therapeutic drugs for the treatment of ASD through the suppression of neuroinflammation.

## 4. The Tripartite Model: A Dynamic Feed-Forward Loop of Brain Aging, Neuroinflammation, and Epigenetics

Building upon the preceding chapters, we propose a tripartite model in which brain aging, neuroinflammation, and epigenetic remodeling interact in a dynamic, self-reinforcing feed-forward loop. This model goes beyond linear or pairwise interactions between these components and conceptualizes them as an interdependent regulatory triangle. Within this framework, each node—aging, inflammation, or epigenetic alteration—can function both as a driver and a consequence of the other two, collectively driving the progression of neurodegenerative processes.

### 4.1. Model Overview: Interdependency and Amplification

In the healthy brain, homeostatic control of inflammation, epigenetic plasticity, and age-associated transcriptional regulation maintains neuronal and glial function. However, with aging, epigenetic drift and loss of chromatin integrity predispose brain cells to aberrant gene expression patterns and increased vulnerability to inflammatory stimuli. In turn, chronic low-grade neuroinflammation further disrupts DNA methylation patterns, alters histone landscapes, particularly in glial cells such as microglia and astrocytes.

These inflammation-induced epigenetic alterations are not merely transient but often persistent, forming maladaptive transcriptional states that impair neurotrophic support and stress resilience. As a result, the accumulation of metabolic stress, ROS, and immune activation further accelerates brain aging. This vicious cycle thus connects the decline in transcriptional plasticity, heightened immune reactivity, and structural chromatin deterioration into a single progressive loop. Importantly, the feed-forward strength intensifies with age, pushing the nervous system from a state of resilience toward irreversible degeneration.

### 4.2. Case Example of the Network Disorder: Neurodegenerative Diseases

#### 4.2.1. Alzheimer’s Disease (AD)

AD exemplifies the complex tripartite feed-forward loop between aging, neuroinflammation, and epigenetic dysregulation. While classical models emphasize Aβ plaques and tau tangles, convergent human and animal studies now show that glial activation, immune remodeling, and chromatin-level alterations are integral to disease progression.

It has been observed that the epigenetic clock is accelerated in AD contexts. In the 3xTg-AD model mouse, the cortex shows a stronger acceleration of the clock than the hippocampus, consistent with region-specific vulnerability [97]. At the DNA methylation level, genome-wide profiling of the superior temporal gyrus has identified hundreds of AD-associated differentially methylated regions, many overlapping poised promoters marked by H3K4me3 and H3K27me3, suggesting aberrant programming of neuronal and immune-associated genes [98]. Single-cell profiling revealed disease-associated microglia and related human microglial states with inflammatory/chemokine programs and *Apolipoprotein E* (*Apo-E*) upregulation. These states display chromatin remodeling at inflammatory loci and upregulation of cytokine/chemokine pathways (e.g., *IL-1β*, *C-C motif chemokine ligand 2* (*CCL2*)), supporting an epigenetically primed innate immune response in AD [36,99,100,101].

At the chromatin level, AD brains show large-scale histone acetylation reconfiguration, including extensive H3K27ac changes at enhancers/promoters and loss of H4K16ac in the lateral temporal lobe; in parallel, *Hdac2* upregulation is linked to repression of synaptic plasticity-related genes and memory impairment. Rather than a uniform global decrease, the acetylation landscape appears imbalanced and redistributed across disease-relevant regions [21,102,103].

A recent study showed that, in mouse models, aging leads to the accumulation of endogenous DNA ligands in microglia (e.g., gDNA and mtDNA), which is transferred to neurons via extracellular vesicles and, by triggering the cGAS–STING pathway, elicits a widespread and sustained neuroinflammatory response that in turn induces neuronal degeneration [104]. This result provides a new possibility for triggering inflammation: endogenous genes generated by aging can not only activate inflammation-related pathways within the cytosol but may also be transferred to other types of cells to elicit inflammatory responses.

**Figure 3:**
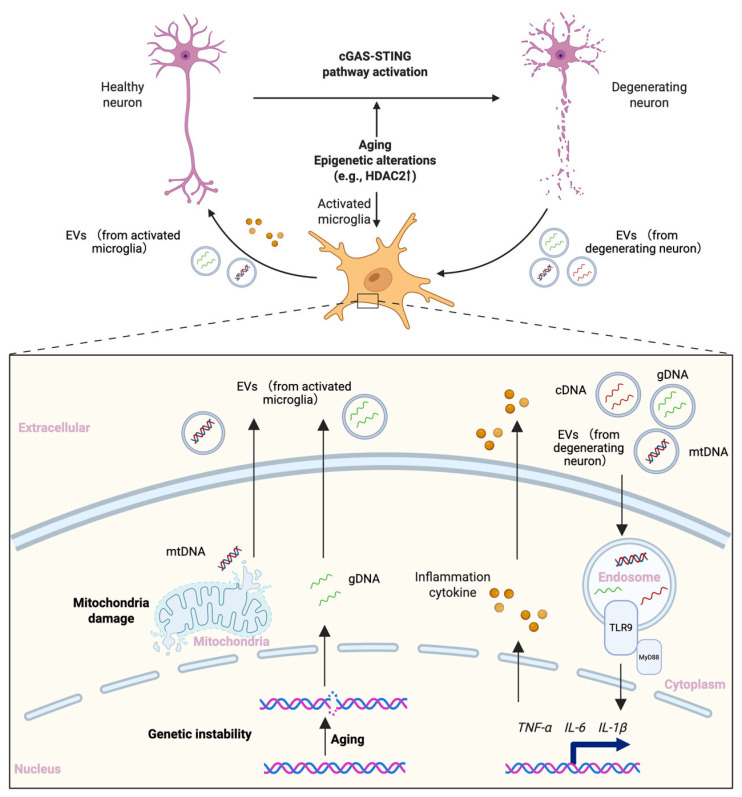
Feed-forward loop linking microglial activation, epigenetic alterations, and neurodegeneration in AD. In AD, the activation of the cGAS-STING pathway driven by aging- and epigenetics-related changes leads to neuroinflammation and neurodegeneration. Endogenous DNA ligands (gDNA, mtDNA, L1 cDNA) leaked from degenerating neurons activate microglia via TLR9 pathway activation, promoting the release of inflammatory cytokines from microglia. Meanwhile, aging and epigenetic alterations also induce the generation of endogenous DNA ligands within microglia, which can be transferred to neurons via extracellular vesicles. This bidirectional exchange exacerbates the inflammatory milieu, further drives neuronal degeneration, and establishes a self-perpetuating feed-forward loop. mRNA: messenger RNA. cDNA: complementary DNA. gDNA: genomic DNA. mtDNA: mitochondrial DNA. EVs: Extracellular vesicles. Created with BioRender.com.

Integrating the pivotal role of epigenetic alterations in aging and neuroinflammation, we sought to explain part of AD pathogenesis with a feed-forward loop framework. As neurons age and undergo epigenetic alterations, gene expression becomes unstable; the resulting endogenous DNA ligands, such as gDNA, mtDNA and L1 cDNA, activate the cGAS–STING pathway, provoking neuroinflammation, neuronal degeneration, and microglial activation with release of inflammatory cytokines (e.g., TNFα, IL-6). Endogenous DNA ligands leaking from degenerating neurons further stimulate microglia, probably through TLR9 [63,105], to secrete cytokines, exacerbating the toxic inflammatory milieu in the brain. Conversely, aging and epigenetic alterations can also drive the accumulation of endogenous DNA ligands within microglia, which can be delivered to neurons via extracellular vesicles, thereby further engaging neuronal cGAS–STING and sustaining a broad inflammatory cascade (Figure 3). Under such chronic neuroinflammatory conditions, the genomic instability driven by aging and epigenetics is further exacerbated, and microglial immune functions become dysregulated, diminishing their protective role against neuroinflammation. On this basis—combined with other pathological processes such as tau protein deposition—irreversible neurodegenerative decline in brain function ultimately occurs.

Together, our model frames AD not merely as a disorder of peptide aggregation, but as a systems level collapse of immune epigenetic homeostasis, wherein aging related epigenetic drift, glial inflammatory priming, and chromatin remodeling act in concert to mutually reinforce disease progression.

#### 4.2.2. Parkinson’s Disease (PD)

Supportive evidence for the tripartite loop model we propose has been confirmed in other neurodegenerative diseases (such as PD and amyotrophic lateral sclerosis (ALS), in addition to AD. Of note is the increase in endogenous DNA ligands induced by aging and epigenetic dysregulation, which activates the cGAS-STING pathway and triggers neuroinflammation.

In PD, misfolded α-syn aggregates in Lewy bodies and neurites, impairing synaptic vesicle trafficking and mitochondrial quality control. The oligomeric/fibrillar species of α-syn disseminate in a prion-like manner, imposing oxidative and genotoxic stress on microglia, which subsequently reprogram astrocytes into neurotoxic states [106,107,108,109]. In addition, aging induces epigenetic alterations (DNA demethylation) at PD risk gene loci (most prominently *Synuclein alpha* (*SNCA*) and *Parkin* (*PARK2*)), resulting in the upregulation of their expression, which further promotes the accumulation of α-syn. Moreover, aging-induced impairment of mitophagy–lysosomal flux and of genome maintenance increase endogenous DNA ligands (e.g., mtDNA, gDNA and L1 cDNA) in the cytosol that triggers cGAS–STING pathway activation [110,111,112,113,114,115,116]. It has been also reported that aggregated α-syn induces an increase of endogenous DNA ligands (e.g., mtDNA and gDNA), activating the cGAS–STING pathway [117]. This pathway activation results in the microglia–astrocyte inflammatory cascade and accelerates nigral degeneration, whereas genetic or pharmacologic attenuation of cGAS–STING signaling has been shown to mitigate the neuroinflammation and pathology of a PD mouse model [118,119,120,121]. Taken together, in PD, the following axis is assumed to exist: “Aging → Epigenetic dysregulation → α-syn aggregation → Organellar and genome stress → Neuroinflammation via cGAS–STING pathway activation → Degeneration of dopaminergic neurons” [110,118,119,120,121,122]. Therefore, the components in this axis could be a promising therapeutic target for the treatment of PD.

#### 4.2.3. Amyotrophic Lateral Sclerosis (ALS)

In the dominant histopathology of ALS, that is, in TAR DNA-binding protein 43 (TDP-43) proteinopathy, TDP-43 plays a crucial role in the induction of genome maintenance defects and mitochondrial stress, which activate innate immune signaling. Loss or abnormal localization of TDP-43 disrupts nuclear RNA/DNA homeostasis and chromatin structure, relaxing heterochromatin around L1 genes to increase their expression while broadly reducing the expression of DNA damage and repair-related genes [123,124]. In parallel, TDP-43 dysfunction drives mtDNA release through the 1-methyl-4-phenyl-1,2,3,6-tetrahydropyridine (MPTP), which is sensed by cGAS to activate STING and type-I interferon programs. This mechanism has been demonstrated in patient-derived iPSC motor neurons and mouse models and is mitigated by genetic or pharmacological pathway inhibition [125,126,127]. In addition, it was previously reported that de-repression of retrotransposons contributes to neurodegeneration even in a Drosophila TDP-43 model, supporting a feed-forward loop between genome instability and neuroinflammation [128,129,130]. Together, these data outline an ALS pathway logic: Nuclear depletion of TDP-43 → chromatin/retroelement instability and mtDNA leakage → cGAS–STING–driven inflammation → motor-neuron degeneration.

Taken all together, the body of evidence spanning AD, PD, and ALS argues strongly for the broad relevance of our proposed tripartite feed-forward loop to brain aging and neurodegeneration. Regarding the putative route in which aging-associated L1 upregulation drives cGAS–STING pathway activation, findings from animal models and human specimens provide substantial indirect support that this mechanism likely operates across multiple neurodegenerative conditions (Table 2). However, to the best of our knowledge, no study in PD or ALS has yet shown that selective suppression of L1 expression directly attenuates cGAS–STING activation in the same model. Establishing this causal linkage—in vivo or in rigorously humanized systems—thus represents an urgent experimental priority for the field.

## 5. Epigenetics-Related Therapeutic Interventions

**Figure 4:**
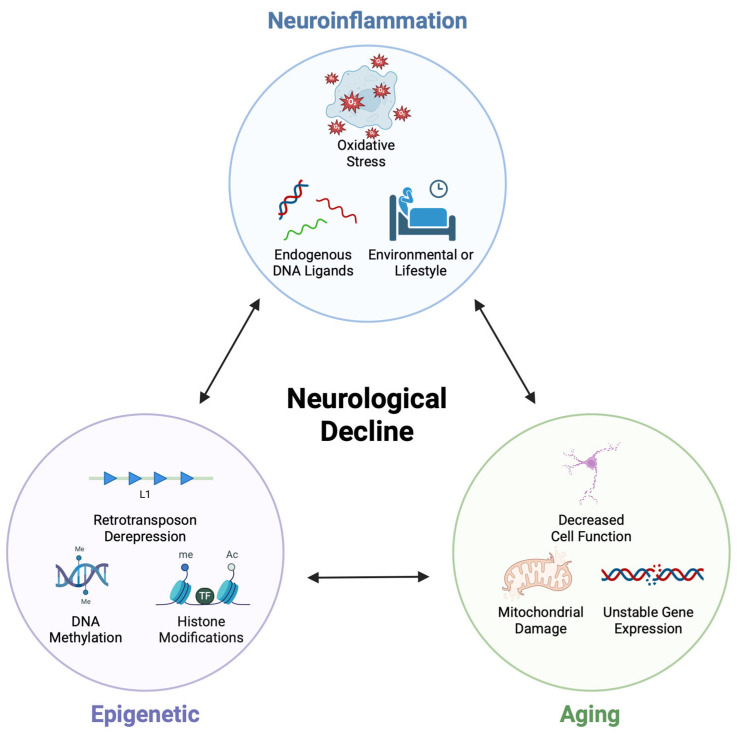
A tripartite feed-forward loop linking aging, neuroinflammation, and epigenetic remodeling. Each node (aging, neuroinflammation, epigenetic alterations) acts both as driver and consequence of the others. Aging induces epigenetic drift and chromatin imbalance that lower inflammatory thresholds; inflammatory signaling remodels enhancer landscapes and histone/DNA marks; epigenetic alterations, including enhancer priming and endogenous DNA ligands/cGAS–STING activation, sustain neuroinflammatory tone. This framework suggests that single-target interventions may be insufficient and supports mechanism-aligned strategies that concurrently dampen inflammatory tone and restore epigenetic plasticity. Created with BioRender.com.

During brain aging, the interplay among epigenetic reprogramming, innate immune activation, and cellular senescence defines a central axis of brain functional vulnerability. We have outlined how intrinsic and extrinsic stressors initiate and propagate this axis through convergent mechanisms. The tripartite model proposed here emphasizes that aging, inflammation, and epigenetic dysregulation form a mutually reinforcing chain that accelerates cognitive decline and heightens susceptibility to neurodegenerative diseases (Figure 4).

Within our tripartite framework, two axes are important. Axis 1 links environmental exposures and lifestyle factors with chronic neuroinflammation to drive epigenetic age acceleration and DNA damage, which induces functional compromise of neural cell types (most notably microglial immune dysregulation) and diminishes cellular resilience to aging. These changes, in turn, accelerate brain senescence and increase vulnerability to neurodegeneration. Axis 2 links aging- and disease-associated gene instability to proteinopathy. Accompanied by increased endogenous DNA ligands (e.g., gDNA, L1 cDNA and mtDNA), cGAS–STING pathway is engaged to amplify neuroinflammation. Targeted interventions along these two axes may therefore provide a rationale for anti-aging and disease-modifying strategies in neurodegenerative disorders.

The timing of the intervention is also important. To determine the optimal timing, it is crucial to leverage cutting-edge technologies such as AI-assisted high-dimensional single-cell profiling (scRNA-seq, scATAC-seq, single-cell methylome) to learn and detect epigenetic and inflammatory markers within the feed-forward loop (e.g., L1 cDNA). This enables early prediction of brain aging trajectories and neurodegenerative progression, contributing to the design of intervention strategies (e.g., modulation of key epigenetic writers/erasers, restoration of redox balance, adjustment of neuroimmune tone). Animal studies indicated that overexpression of *Tet2* or inhibition of *Hdac2* can enhance NSC activity and improve the functions of the aged brain [16,143]. In parallel, given that loss of epigenetic silencing elevates L1 mRNA expression, which is reverse-transcribed into cDNA and can trigger cGAS–STING pathway activation, to prevent cDNA production by reverse-transcriptase inhibitors represents a feasible therapeutic measure [144].

Despite abundant animal data and a small number of early clinical observations supporting our proposed model, deeper validation using human cells and tissues is necessary to account for interspecies differences. We believe there are two paths we can take to achieve this. First is to leverage patient-derived iPSC brain organoids that can recapitulate human-specific phenotypes attributable to human genetic backgrounds [145,146,147]. Second is to construct spatiotemporal atlases of human brain aging–inflammation–epigenetic alterations—analogous to recent works in human brain development [148]. Although conventional single-cell assays have not been able to present information of histone modification changes at single-cell level, emerging platforms provide a new possibility: The Epitope-based Time-of-Flight Mass Spectrometry (EpiTOF) method simultaneously quantifies multiple histone modifications/epigenetic enzymes and cell lineage markers at single-cell resolution to derive cell type-specific “epigenetic fingerprints” [149]. Spatial-DMT (DNA Methylome and Transcriptome) simultaneously profiles DNA methylation and transcription across tissue sections, mapping genome-wide DNA methylation and elucidating the spatial coupling of epigenetic states and transcription [150].

However, several clear limitations still constrain the application of these two technologies. EpiTOF depends heavily on antibody specificity, limiting cross-laboratory comparability. Spatial-DMT currently operates at pixel, rather than true single-nucleus resolution. Accordingly, optimizing assay process and improving quantitative precision, while establishing standardized workflows and reference datasets, will be decisive for building reliable, human-anchored spatiotemporal maps of aging, inflammation, and epigenetic regulation.

Suppression of neuroinflammation is increasingly recognized as an effective anti-aging strategy [151]. Given that our above-mentioned Axis 2 converges on cGAS–STING pathway as a principal route to neuroinflammation, we view pharmacologic inhibition of the cGAS–STING pathway as a practical therapeutic direction. To that end, we summarize in Table 2 preclinical and clinical attempts to dampen cGAS–STING signaling across aging, AD, PD, ALS, and other neurodegenerative disorders. Although human data on cGAS–STING inhibition remain limited and largely in early-phase, encouragingly, open-label phase IIa pilot trial (NCT04552795) enrolling participants with MCI/early AD, oral lamivudine (3TC, a reverse transcriptase inhibitor) administration for 24 weeks was safe and well tolerated and was associated with reductions in multiple CSF biomarkers of neurodegeneration and neuroinflammation. An increase in the plasma Aβ 42/40 ratio—typically interpreted as consistent with lower amyloid burden was observed, although the study did not directly quantify brain plaque load [135]. Looking forward, we anticipate and encourage more clinical evaluations of cGAS–STING pathway suppression, pathway modulation, and disease modification.

Ultimately, current findings on neurodegeneration and anti-aging support the validity of our proposed tripartite model, in which epigenetic alterations mediate the crosstalk between neuroinflammation and brain aging. Further exploration of this model—and elucidation of the mutual influences among its three components—will have substantial research and translational value for therapies targeting neurodegenerative diseases and brain aging.

## Figures and Tables

**Table 1:** Epigenetic alterations in aging of CNS cells.

Cell Type	Epigenetic Alterations	Outcome	References
NSC	LaminB1 expression progressively declines	Silencing of neurogenic genes	[6,7]
	Loss of *Setd1a* (H3K4me)	Promotes NSC activation	[8]
	Downregulation of *Setd8* expression	Upregulation of quiescence-associated genes, inhibition of NSC proliferation	[9]
	Downregulation of *Tet2* expression	Impairing adult neurogenesis	[10]
Neuron	α-tubulin undergoes hyperacetylation	Loss of dendritic arbors and spines	[11]
	Increased expression of Hdac2	Synaptic loss and reduced plasticity	[12]
Astrocyte	Increased expression of HDAC7	Contributes to tau accumulation and degeneration of neurons	[13]
Oligodendrocyte/OPC	Reduced expression of *Dnmt1*	Global DNA hypomethylation was observed, reduced remyelination efficiency and impaired OPC differentiation	[14]
Microglia	Downregulation of *Sirt1* expression	Upregulation of *Il-1β* expression	[15]
	Increased expression of TET2	Microglial activation	[16]

**Table 2:** Studies of cGAS-STING pathway inhibitor in aging and neurodegenerative diseases.

Disease	Conclusions	Models	Strategy	Reference
Aging	Reduced inflammation associated with aging. Resulting in improved tissue function.	Aging mice	H-151, si-STING	[56]
AD	Microglia transition from a harmful phenotype to a protective phenotype. Decreased expression of *IL-6*, *Il-1β*, and *Tnf-α*. Increased expression of *Arg1* and *Fizz1*. Reduced degree of aging.	APP/PS1 mice	H-151, si-STING	[131]
	Reduced brain inflammation and microglial synaptic phagocytosis. Significantly improved Aβ burden, tau phosphorylation, and cognitive impairment.	App^NL-G-F^/hTau-double KI mice	H-151	[132]
	Increased Aβ clearance. Suppression of neurotoxic A1 astrocytes. Decreased expression of *Ifn-β*, *Il-6*, *Tnf-α*, and *Il-1α*. Enhanced phagocytic activity of microglia. Alleviation of cognitive impairment and Aβ pathological changes.	5×FAD mice	H-151	[133]
	Reduced of cleaved caspase 3.	PLD3 knockout SH-SY5Y cells	H-151	[134]
	Decreased GFAP in cerebrospinal fluid [reduced neuroinflammation). Elevated Ab42/40 ratio in plasma (reduced plaque burden in the brain).	Human	Lamivudine (3TC)	[135]
	Reduced expression of single-stranded DNA. Reduced DNA damage. Reduced neuronal death. Increased expression of *PI16* and *ADAMDEC1.* reduction of Aβ deposition p-tau and K63-linked ubiquitin-positive tau	3D neural spheroids generated from late-onset AD patient fibroblasts via direct neural reprogramming technology	3TC	[136]
	Reduced tau phosphorylation, inflammation, neuronal death, and hippocampal atrophy. Alleviated motor deficits (Rotarod test) and improved short-term memory (Y-maze test). Inhibited the insertion of L1.	P301S mice	3TC	[137]
	Improved cognitive function. Reduced inflammation and anxiety. Reduced Iba1 and GFAP expression.	rTg4510 mice	3TC	[138]
	Enhanced the neuronal *Mef2c* transcriptional network. Restored synaptic integrity, plasticity, and memory.	P301S transgenic mice	TDI-6570	[65]
	Reduced of p21 positive cells.	Mouse microglia cells	TDI-6570	[139]
	Restricted Aβ deposition. Alleviated neuroinflammation. Reduced neuronal damage. Improved cognitive behavioral.	5xFAD; cGAS^fl/fl^; Cx3cr1^+/−^ mice	STING deficiency	[140]
PD	Reduced interferon expression in the striatum. Treatment of motor dysfunction, pathological α-synuclein deposition, and dopaminergic neuron loss.	Sting^gt^ mice	STING deficiency	[115]
	Attenuated PD-associated behavioral phenotypes. Reduced loss of TH-positive neurons. Decreased the number of activated microglia. Lowered levels of factors associated with cGAS-dependent inflammation.	MPTP PD mice	RU.521	[141]
	Reduced expression of *Ifn-β*, *Tnf-α*, *Il-1β*, and *Il-6*. Decreased expression of Nlpr3 and caspase-1. Alleviated neuroinflammation associated with MPTP neurotoxicity. Protected substantia nigra striatal dopaminergic neurons from degeneration. Lowered levels of reactive astrocytes.	MPTP PD mice	C-176	[120]
ALS	Reduced of IFN-I, ISGs, pro-inflammatory, and chemokines genes. Improved motor functions.	SOD1 ALS mice	C-176, H-151, RU.521	[125]
	Reduced the expression of *Il-1β*, *Il-6*, *Tnf*, *Mx1*, *Infb 1*. Decreased neuronal loss.	TDP-43 mutant mice	H-151	[123]
	Preventing motor neuron death.	Human iPSC-derived motor neurons		
	Reduced levels of *TNF-α*, *IL-6* and *CXCL10*. Suppressed inflammatory responses.	Human iPSC-derived neurons	H-151, RU.521	[124]
		C9orf72 mice		
	Reduced of ISGs expression	C9orf72 knockout mice	H-151	[142]

*Arg1*: *Arginase 1*; *Fizz1*: *Resistin like beta*; GFAP: Glial fibrillary acidic protein; *PI16*: *Peptidase inhibitor 16 ADAMDEC1*: ADAM like decysin 1; Nlpr3: NLR family pyrin domain containing 3; IFN-I: Interferon type I; ISGs: Interferon stimulated genes; *Mx1*: *Interferon-induced GTP-binding protein Mx1*; *Infb 1*: *Interferon beta 1*; *CXCL10*: CXC Motif chemokine ligand 10.

## Data Availability

Not applicable.
